# Beyond the quadriceps tendon: The rectus femoris as a distinct autograft for knee ligament reconstruction—A narrative review

**DOI:** 10.1002/jeo2.70862

**Published:** 2026-07-30

**Authors:** Bernardo Garcia Barroso, Marcelo Cabral Fagundes Rêgo, Márcio Cabral Fagundes Rêgo, Felipe Silveira Martins, Sergio Marinho de Gusmão Canuto, Marcos Barbieri Mestriner, Carlos Eduardo Franciozi, Camilo Partezani Helito

**Affiliations:** ^1^ Instituto de Ortopedia e Traumatologia Vitória Apart Hospital Serra Espírito Santo Brazil; ^2^ Grupo de Joelho, Hospital Memorial São Francisco Natal Rio Grande do Norte Brazil; ^3^ Grupo do Joelho, Clínica de Ortopedia e Traumatologia Balneário Camboriú Santa Catarina Brazil; ^4^ Hospital da Unimed Balneário Camboriú Santa Catarina Brazil; ^5^ Ortoclínica Hospital de Ortopedia Maceió Alagoas Brazil; ^6^ Departamento de Ortopedia e Traumatologia Grupo de Joelho, Santa Casa de São Paulo São Paulo São Paulo Brazil; ^7^ Departamento Ortopedia da Escola Paulista Medicina UNIFESP São Paulo Brazil; ^8^ Grupo de Joelho, Instituto de Ortopedia e Traumatologia, Hospital das Clínicas HCFMUSP, Faculdade de Medicina Universidade de São Paulo São Paulo São Paulo Brazil

**Keywords:** anterior cruciate ligament reconstruction, autograft, biomechanics, graft selection, knee ligaments, quadriceps tendon, rectus femoris tendon

## Abstract

**Purpose:**

This narrative review synthesises the anatomical, biomechanical, and clinical evidence on the rectus femoris tendon as an autograft for knee ligament reconstruction.

**Methods:**

A literature search was conducted in PubMed and Google Scholar up to June 2026 with no language restrictions. A total of 35 articles describing the rectus femoris tendon as a graft for knee ligament reconstruction were included.

**Results:**

Cadaveric studies demonstrated consistent morphometric dimensions, with a mean tendon length of approximately 30 cm and graft diameters ranging from 8 to 11 mm. Biomechanical testing across four independent cadaveric studies showed that the doubled rectus femoris provides load‐to‐failure within the range of established anterior cruciate ligament grafts. Multiple harvesting techniques have been described, using the identification of a fat layer between the rectus femoris and the intermediate layer as the primary landmark. The graft has been applied to isolated anterior cruciate ligament reconstruction, combined anterior cruciate ligament and anterolateral or anteromedial ligament reinforcement, posterior cruciate ligament reconstruction, and revision surgery. Early clinical series reported functional outcomes comparable to hamstring tendon autografts, with low donor‐site morbidity. Isokinetic evaluation showed no difference in extensor performance between rectus femoris and hamstring tendon groups at six months. Intraoperative complications included capsular violations and premature graft amputations, concentrated in initial cases and without clinical repercussions. The current clinical evidence is limited to level III and IV studies, with a maximum mean follow‐up of approximately 3.5 years in revision settings and 3 years in primary reconstruction, demonstrating improvement in patient‐reported outcomes and knee stability.

**Conclusion:**

The rectus femoris tendon is a viable autograft for knee ligament reconstruction, with consistent anatomy, adequate biomechanical properties, and early clinical results comparable to hamstring tendon autografts. It should be considered in individualised graft selection.

**Level of Evidence:**

Level V.

## Graphical abstract

Bottom line: Current evidence supports the rectus femoris tendon as a viable autograft for knee ligament reconstruction, with adequate biomechanical properties, low donor‐site morbidity, and clinical outcomes comparable to hamstring autografts.

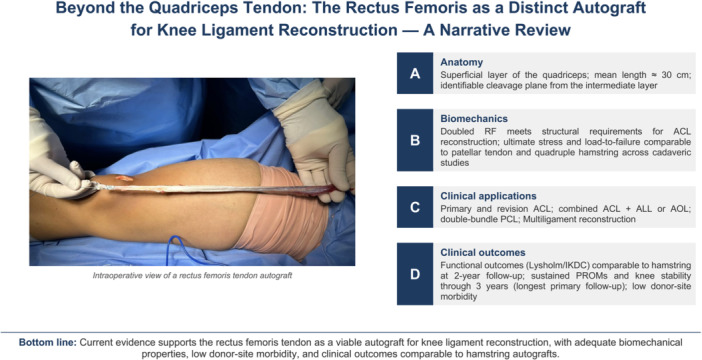

**Abbreviations**
ACL: anterior cruciate ligament
ALL: anterolateral ligament
AMOL: anteromedial oblique ligament
HT: hamstring tendon
ITB: iliotibial band
LSI: limb symmetry index
NR: not reported
ns: not significant
PASS: patient‐acceptable symptom state
PLT: peroneus longus tendon
PT: patellar tendon
QT: quadriceps tendon
RF: rectus femoris
SRF: single rectus femoris

## INTRODUCTION

Autografts remain the standard for anterior cruciate ligament (ACL) reconstruction, with bone–patellar tendon–bone, hamstring tendon (HT), and quadriceps tendon being the most commonly used options [39, 41]. Each presents well‐documented advantages and limitations, and no single graft meets all requirements regarding biomechanical performance, donor‐site morbidity and functional outcomes [35, 41]. This has motivated the exploration of additional graft sources [13].

Within the quadriceps tendon complex, the rectus femoris (RF) constitutes the most superficial layer and can be individually harvested through a natural cleavage plane [12, 18, 31]. Unlike full‐ or partial‐thickness quadriceps tendon grafts, the RF is a homogeneous tendinous structure with parallel collagen fibres, an average length of approximately 30 cm, and predictable cross‐sectional dimensions [3, 18]. The harvest requires no bone block and standard ACL reconstruction instrument set, including a rigid tendon stripper, and it preserves the deeper layers of the extensor mechanism entirely [2, 31]. These features make the RF a distinct graft option rather than a variation of the quadriceps tendon [26].

Since its initial description, the RF has been applied to progressively complex clinical scenarios, including isolated ACL reconstruction, combined ACL and anterolateral or anteromedial ligament reinforcement, double‐bundle posterior cruciate ligament reconstruction and revision ACL surgery [2, 10, 19, 33, 34]. Biomechanical studies have demonstrated that the RF, when prepared in folded configurations, achieves mechanical properties equivalent to those of the patellar tendon [20, 27]. Early clinical series have reported satisfactory functional outcomes with low donor‐site morbidity [34], and comparative studies have shown results comparable to HT autografts [11, 32].

The purpose of this narrative review is to synthesise the anatomical, biomechanical and clinical evidence on the rectus femoris tendon as an autograft for knee ligament reconstruction.

## METHODS

A literature search was conducted in PubMed and Google Scholar from inception to June 2026, with no language restrictions. Search terms combined graft‐specific keywords ('rectus femoris', 'rectus femoris tendon', 'superficial quadriceps tendon', 'superficial layer quadriceps tendon', and 'superficial layer of the quadriceps') with terms related to knee ligament reconstruction ('anterior cruciate ligament', 'ACL', 'posterior cruciate ligament', 'PCL', 'anterolateral ligament', 'ALL', 'knee ligament reconstruction', 'graft', 'autograft', 'harvest', 'anatomy', and 'biomechanics'). Reference lists of all included articles were manually screened for additional relevant studies.

Studies were included if they described the rectus femoris tendon as a graft for knee ligament reconstruction, or addressed its anatomy, biomechanics, harvesting technique, clinical applications, or donor‐site morbidity. Studies were excluded if they (1) addressed full‐thickness or partial‐thickness quadriceps tendon grafts without specific reference to the rectus femoris or superficial layer; (2) reported rectus femoris injuries, strains, ruptures, or avulsion fractures; or (3) were duplicate reports of the same patient cohort.

A total of 35 articles addressing the rectus femoris tendon were included in the final synthesis. This study was designed as a narrative review. The characteristics of all 35 included studies, grouped by study type (anatomical, biomechanical, surgical technique, clinical and editorial) with study design, sample size, focus, follow‐up, and key findings, are summarised in Supporting Information: Table S1.

### ANATOMY

**Figure 1:**
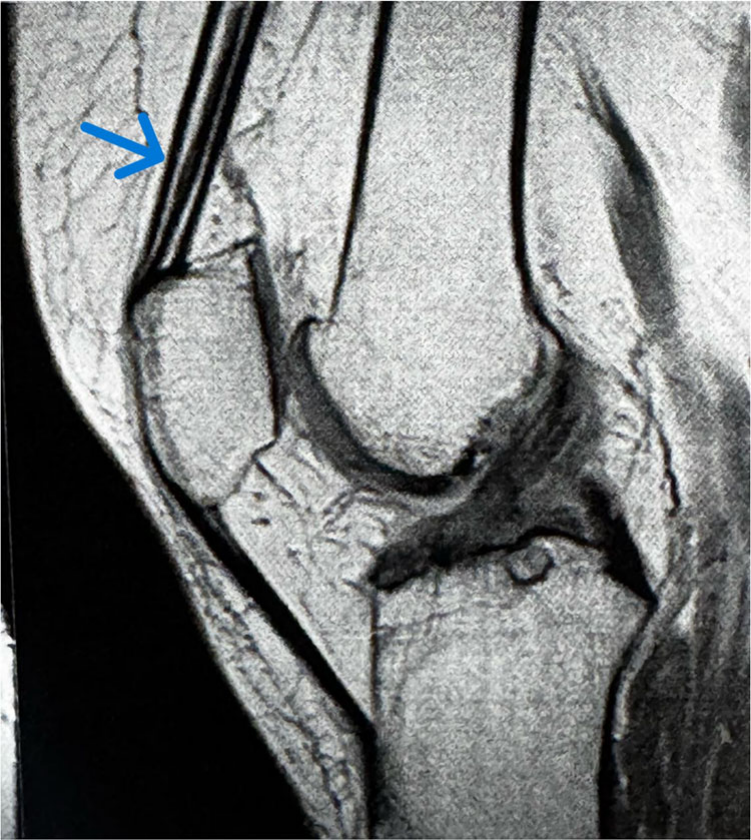
Anatomy. Sagittal magnetic resonance image of the knee showing the three‐layered architecture of the quadriceps tendon. The superficial rectus femoris layer (blue arrow) is clearly delineated from the intermediate and deep layers by a thin hyperintense fat plane.

The quadriceps tendon is a multilayered structure, with the rectus femoris forming its most superficial layer [12, 40]. A recent meta‐analysis of 1066 cadaveric specimens confirmed that trilaminar and quadrilaminar configurations occur in nearly equal proportions, demonstrating greater architectural variability than classically described [25]. Despite this variability in the deeper layers, the rectus femoris consistently forms an independent superficial lamina that can be separated through a natural cleavage plane [12] (Figure 1).

**Figure 2:**
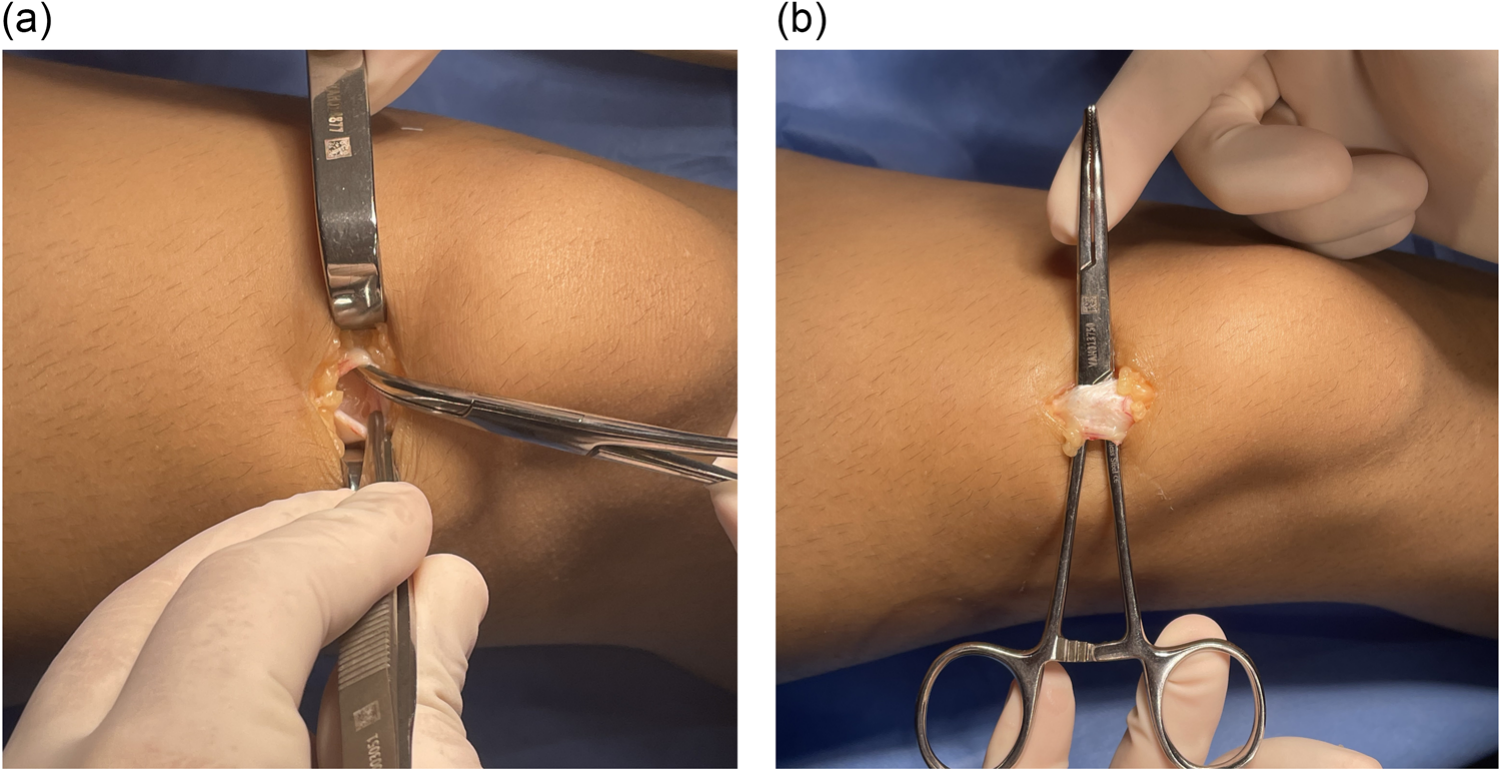
Intraoperative view of a right knee. (a) The surgical plane between the superficial rectus femoris lamina and the intermediate layer of the quadriceps tendon is identified approximately 3 cm proximal to the superior pole of the patella. (b) Two parallel longitudinal incisions are made in the superficial layer, creating a 10‐mm‐wide graft strip. The rectus femoris is elevated from the underlying quadriceps tendon with a haemostatic forceps, confirming the correct dissection plane and graft width.

Marot et al. identified two consistent fusion zones between the rectus femoris and the deeper quadriceps layers: a distal confluence at a mean of 22 mm and a proximal confluence at 58 mm above the upper pole of the patella, defining a 36‐mm segment free of deep attachments [18]. This anatomical window corresponds to the harvest area described in surgical techniques, where a longitudinal skin incision of 3–4 cm is made approximately 3 cm proximal to the superior patellar pole [3, 31]. A natural fat layer between the rectus femoris tendon and the intermediate layer serves as the primary landmark for correct plane identification [3, 18, 31]. Two parallel longitudinal incisions are then made on the rectus femoris tendon, and a clamp passed between them confirms both the correct dissection plane and the graft width [3] (Figure 2). If digital separation between the superficial and intermediate layers cannot be easily achieved, the dissection plane should be reassessed [18].

For cadaveric demonstration of the anatomical plane between the rectus femoris and the vastus intermedius, the reader is referred to Figure 2 in Huber et al. [10].

Cadaveric and clinical data have provided consistent morphometric information on the rectus femoris tendon (Table 1). Iriuchishima et al. first reported a mean RF tendon length of 27.3 cm and a narrowest width of 15.3 mm in 16 formalin‐fixed cadaveric knees [12]. Marot et al. reported a mean tendon length of 30.1 cm in 10 cadaveric specimens, with graft diameters of 8.8 mm in double‐strand, 9.9 mm in triple‐strand, and 11.4 mm in quadruple‐strand configurations, and post‐folding graft lengths of 15.1, 9.8 and 7.4 cm, respectively [18]. In a separate cadaveric study of 16 knees using a triple‐strand configuration, Thamrongskulsiri et al. reported a mean graft diameter of 9.7 mm and a post‐folding length of 9.3 cm, with no significant differences between sexes [37]. These cadaveric dimensions were confirmed by Barroso et al. in a prospective multicenter series of 211 patients, in which the mean tendon length was 30.8 cm, with mean diameters of 8.1 mm in double‐strand and 9.3 mm in triple‐strand configurations [3]. In the triple‐strand preparation, a graft diameter below 8 mm was observed in only 1.4% of cases [3]. Height was identified as the strongest anthropometric predictor of tendon length, suggesting that preoperative planning can be individualised based on patient characteristics [3].

**Table 1:** Morphometric characteristics of the rectus femoris tendon.

	Iriuchishima et al. [12]	Marot et al. [18]	Thamrongskulsiri et al. [37]	Barroso et al. [3]
Year	2012	2025	2025	2026
Study Type	Cadaveric	Cadaveric	Cadaveric	Clinical
*N*	16 knees	10 specimens	16 knees	211 patients
Preservation	Formalin	Fresh‐frozen	Thiel	NA
Tendon length (cm)	27.3 ± 1.6	30.1 ± 3.0	28.9 ± 1.0	30.8 ± 3.4
Narrowest width (mm)	15.3 ± 2.9	20.1 ± 3.8	NR	NR
Harvest strip (mm)	NA	10	8	10–12
Doubled diameter (mm)	NA	8.8 ± 0.8	NA	8.1 ± 0.6
Tripled diameter (mm)	NA	9.9 ± 1.0	9.7 ± 0.7	9.3 ± 0.7
Tripled diameter < 8 mm (%)	NA	NR	NR	1.4

*Note*: Values are mean ± SD.

Abbreviations: NA, not applicable; NR, not reported; SD, standard deviation.

### BIOMECHANICS

The biomechanical properties of the rectus femoris tendon have been evaluated across six independent studies. Zhu and Zhang demonstrated that the single‐strand rectus femoris has tensile properties, including elastic modulus, maximum load, and ultimate stress, comparable to those of the native anterior cruciate ligament [42]. Chivot et al. tested each individual layer of the quadriceps tendon in its central and medial portions. The superficial layers, which represent the closest anatomical equivalents to the rectus femoris graft, exhibited ultimate stress superior to the iliotibial band but lower than the patellar tendon, with values in the range of those reported for two‐strand hamstrings [4].

Subsequent cadaveric studies evaluated the biomechanical properties of the doubled rectus femoris tendon graft. Pineda et al. reported comparable ultimate stress between the doubled rectus femoris and the patellar tendon (46.4 vs. 52.9 MPa, *p* = 0.18) despite lower load‐to‐failure (886 vs. 1279 N, *p* < 0.001), suggesting that intrinsic tissue strength is the more relevant measure of graft suitability [27]. Lopes et al. compared seven autograft configurations and found no significant difference in ultimate load among the doubled rectus femoris (1714 ± 56 N), the patellar tendon (1735 ± 136 N), and the quadruple‐strand hamstring (1684 ± 81 N) [15]. Mestriner et al. evaluated the rectus femoris in configurations designed for combined ACL and anterolateral reconstruction [20]. The doubled rectus femoris (1978 ± 338 N) demonstrated ultimate tensile strength equivalent to the patellar tendon (1824 ± 557 N), with a larger cross‐sectional area (39.8 vs. 29.6 mm^2^) and comparable ultimate stress [20]. The single‐strand rectus femoris also showed properties comparable to the iliotibial band for lateral extra‐articular reinforcement [20]. In a separate cadaveric study comparing three graft sources, Egiazaryan et al. found that the superficial quadriceps tendon layer (the rectus femoris equivalent) had the highest ultimate load (1043 ± 4 N), stiffness (156 ± 13 N/mm) and elastic modulus (94.5 ± 8.6 MPa), significantly exceeding both the full‐thickness quadriceps tendon (728 ± 39 N; 84 ± 15 N/mm; 52.8 ± 9.4 MPa) and the peroneus longus tendon (908 ± 74 N; 101 ± 12 N/mm; 78.2 ± 9.2 MPa) (all *p* < 0.001) [8]. The observation that the isolated superficial layer outperformed the full‐thickness construct is counterintuitive, but may reflect a higher density of longitudinally oriented parallel collagen fibres within the rectus femoris tendon when isolated from the deeper, more heterogeneous quadriceps layers.

The dissociation between ultimate stress (US) and load‐to‐failure (UL) reported by Pineda et al. [27] warrants consideration. While ultimate stress reflects the intrinsic material quality of the tissue and was comparable to the patellar tendon, load‐to‐failure is a structural property that depends on the cross‐sectional area and width of the harvested strip. Therefore, ultimate stress may be the more clinically relevant parameter, as graft diameter can be adjusted by changing the folding configuration. The substantial variability in load‐to‐failure values across studies (886 N in Pineda et al. [27] vs. 1,714 N in Lopes et al. [15] vs. 1,978 N in Mestriner et al. [20]) likely reflects differences in donor age (30 vs. 35 vs. 49 years), graft width, fixation methods (serrated clamps vs. interference screws with polyurethane blocks vs. freeze clamps), and testing protocols rather than inconsistencies in tissue quality.

These findings, consistent across independent laboratories, indicate that the doubled rectus femoris provides load‐to‐failure within the range of conventional ACL grafts (Table 2).

**Table 2:** Biomechanical properties of the double rectus femoris tendon graft.

	Pineda et al. [27]	Gadelha Lopes et al. [15]	Mestriner et al. [20]	Egiazaryan et al. [8]
Year	2025	2026	2026	2026
*N* (RF specimens)	7	6	8	8
Specimen	Fresh	Fresh	Fresh‐frozen	Fresh
Mean donor age (years)	30 ± 2	35 ± 5	49 (36–64)	50 ± 5
RF configuration	Doubled	Doubled	Doubled	8 mm (doubled or tripled)
Comparator(s)	PT	PT, QT, HT, PLT, ITB	PT, SRF, ITB	QT, PLT
Fixation method	Serrated clamps	IS + PU blocks	Freeze clamps	Wave clamps
RF ultimate load (N)	886 ± 52	1714 ± 56	1978 ± 338	1043 ± 24
RF ultimate stress (MPa)	46.4 ± 10.5	NR	51 ± 12	NR
Main finding	UTS comparable to PT despite lower UL	UL comparable to PT and 4xHT	UL and UTS comparable to PT	RF superior to QT and PLT

*Note*: Values are mean ± SD.

Abbreviations: HT, hamstring tendon; IS, interference screw; ITB, iliotibial band; NR, not reported; PLT, peroneus longus tendon; PT, patellar tendon; PU, polyurethane; QT, full‐thickness quadriceps tendon; SRF, single rectus femoris; UL, ultimate load; UTS, ultimate tensile stress; SD, standard deviation.

### HARVESTING TECHNIQUE

**Figure 3:**
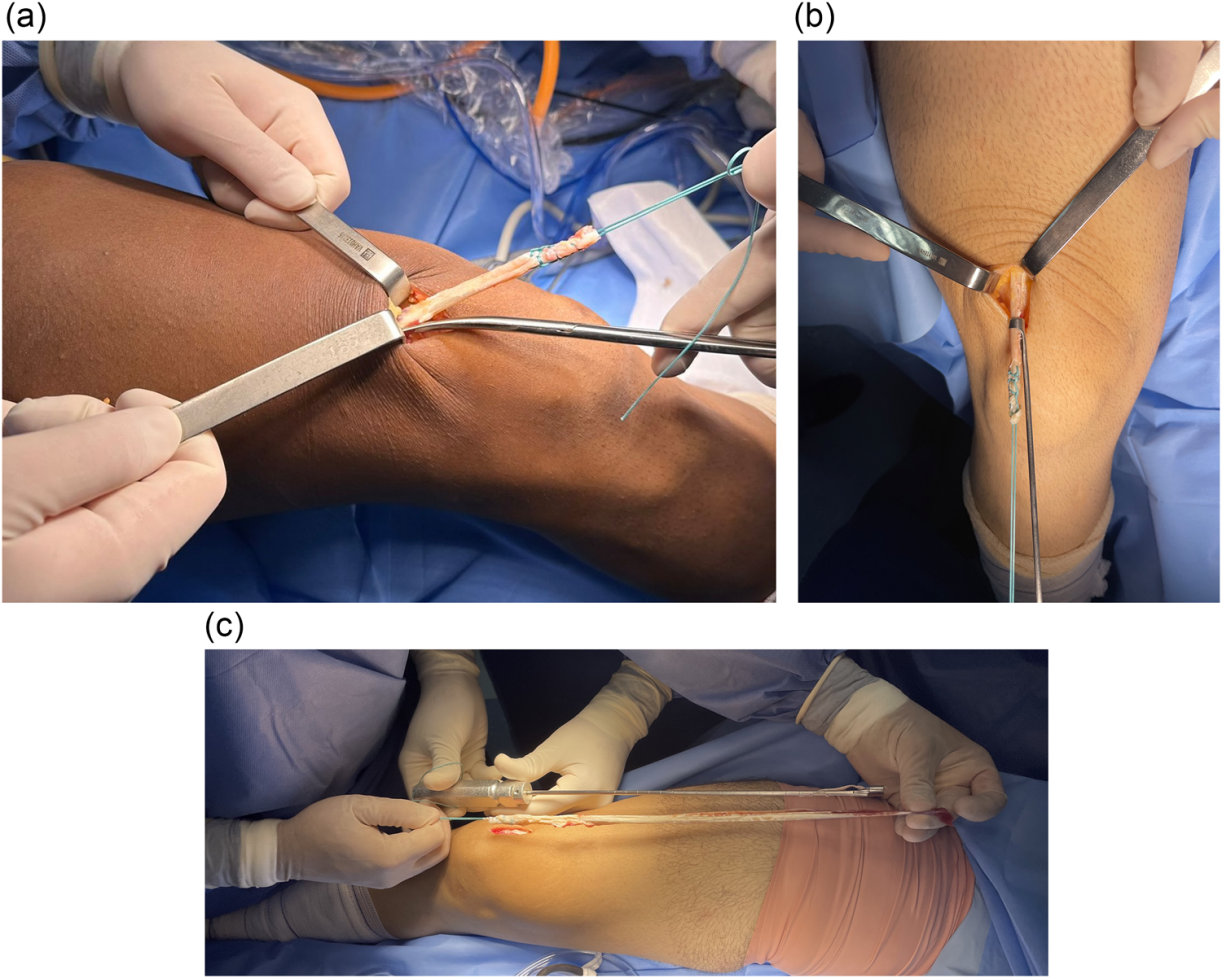
Rectus femoris tendon harvest sequence (right knee). (a) The distal free end of the graft is whipstitched with nonabsorbable sutures. The dissection is extended proximally for approximately 8 cm using Metzenbaum scissors. Complete mobilisation of the graft from the remaining quadriceps layers is confirmed before stripper advancement. (b) A closed tendon stripper is directed toward the anterior superior iliac spine and advanced proximally with the knee in 20 degrees of flexion. (c) The harvested rectus femoris tendon graft.

Several harvesting techniques have been described for the rectus femoris tendon since 2022 [14, 23, 28, 29, 31, 38]. These techniques differ in skin incision orientation, direction of harvest, and type of tendon stripper. The first description used a distal‐to‐proximal approach with a closed tendon stripper [31]. Later techniques introduced transverse skin incision approach [28], triple‐strand configurations [38], tactile confirmation of the dissection plane before stripper advancement [29] and bidirectional harvesting for greater control [14]. However, the fundamental principles are the same across all techniques: identification of the natural cleavage plane between the rectus femoris and the intermediate layer and preservation of the deeper quadriceps layers. In the largest published clinical study, with 211 ACL reconstructions [3], the following key steps for safe harvest were identified: (1) initiating dissection with the knee flexed at 90° to facilitate anatomical identification; (2) performing two parallel scalpel incisions approximately 3 cm proximal and 3 mm deep to define the intended graft width (10–12 mm); (3) identifying the natural cleavage plane with the aid of a Kelly clamp and releasing the distal tendon close to the superior pole of the patella; (4) passing sutures before continuing the proximal sharp dissection with Metzenbaum scissors; (5) confirming at least 7–8 cm of mobilised tendon with complete free proximal–distal excursion within the dissection plane prior to stripper use; and (6) completing the harvest with a rigid tendon stripper directed toward the anterior superior iliac spine, with the knee maintained between 0° and 20° of flexion [3] (Figure 3).

### CLINICAL APPLICATIONS

**Figure 4:**
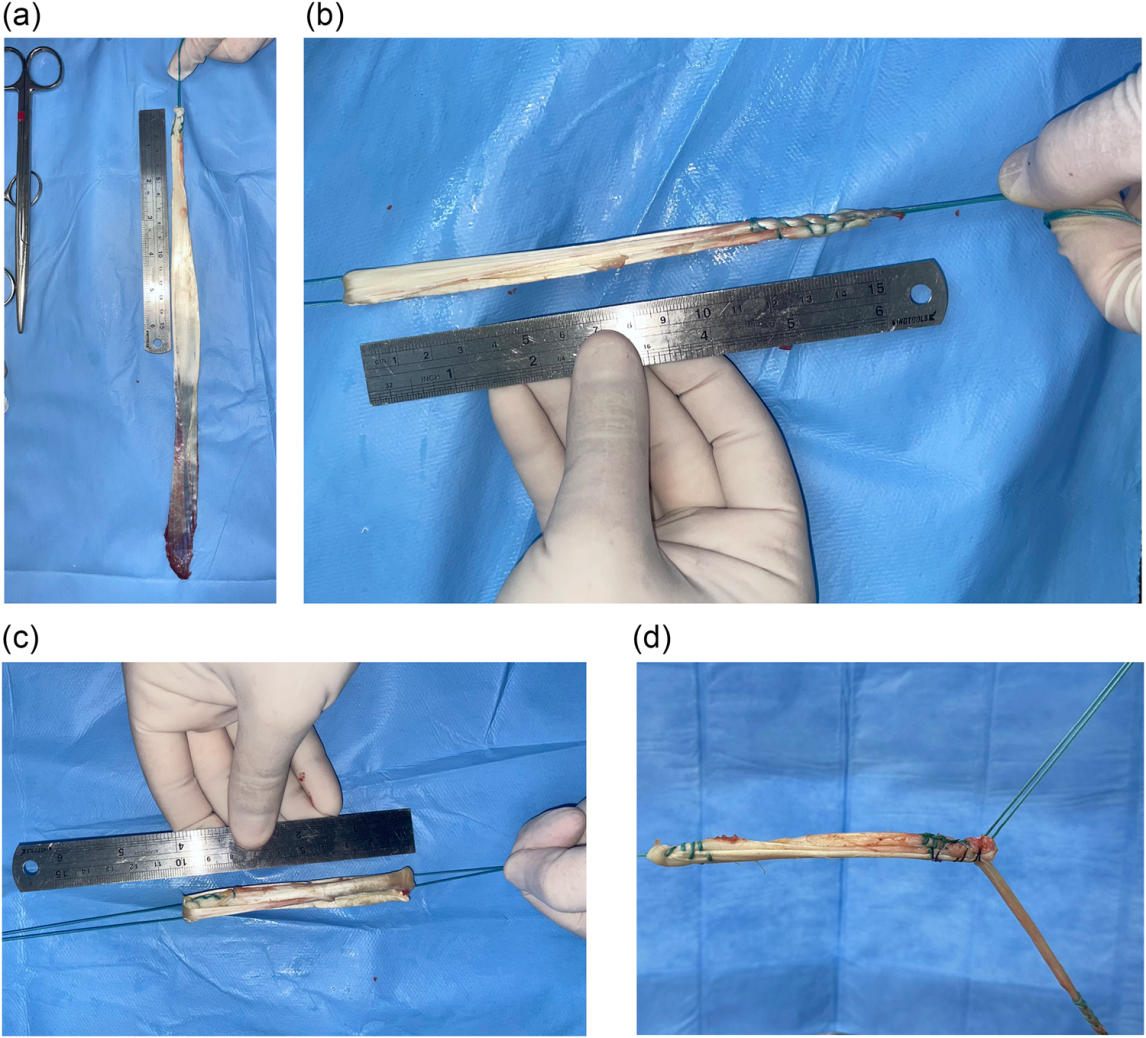
Graft configuration options. (a) The harvested rectus femoris tendon before preparation, measuring 31 cm in total length. (b) Double‐strand configuration for isolated anterior or posterior cruciate ligament reconstruction. (c) Triple‐strand configuration for cases requiring increased graft diameter. (d) Combined configuration: the distal portion is folded to form a double‐strand graft for anterior cruciate ligament reconstruction, while the proximal remainder is used as a single strand for anterolateral or anteromedial ligament reinforcement.

The length of the rectus femoris tendon allows multiple graft configurations for ACL reconstruction (Figure 4). In a double‐strand preparation, the graft can be used for isolated ACL reconstruction [34]. In a triple‐strand configuration, adequate diameter is achieved even in patients with thinner tendons [3], and some studies have utilised a four‐strand configuration [10, 16]. The RF tendon graft can also be used for combined ACL and anterolateral ligament reconstruction: a single strand is used for anterolateral ligament reconstruction (Figure 4d) [2, 7, 16, 24, 36]. The same principle has been applied for combined ACL and anteromedial reinforcement with anterior oblique ligament reconstruction [33]. This approach allows combined intra‐ and extra‐articular reconstruction with one graft while preserving the hamstrings and the deeper quadriceps layers.

The rectus femoris has also been used beyond ACL reconstruction. For double‐bundle posterior cruciate ligament reconstruction, two techniques have been described: one using the RF tendon alone in a Y‐shaped configuration [19], and another combining the RF with the semitendinosus tendon [5]. A composite graft combining a patellar bone block, partial‐thickness quadriceps tendon, and the RF tendon has also been described for combined revision ACL and anterolateral reconstruction, with the aim of optimising tibial fixation through bone‐to‐bone healing in cases with tunnel widening [21].

The rectus femoris tendon is also particularly valuable in multiligament knee injuries, especially in regions where allograft availability is limited or cost‐prohibitive. Its use expands the autograft options available to the surgeon, helping to preserve other commonly harvested graft sources while still providing sufficient tissue for complex reconstructions involving multiple ligaments [9].

### CLINICAL OUTCOMES

Early clinical data on the rectus femoris graft are limited but consistent (Table 3). Rêgo et al. reported a mean Lysholm score of 97.1 at 18 months in a series of 80 primary ACL reconstructions, with all patients reaching the patient‐acceptable symptom state (PASS), defined as the threshold of symptoms beyond which patients consider their condition to be satisfactory [34]. Dhariwal et al. compared 80 primary ACL reconstructions (40 RF vs. 40 HT) and reported equivalent IKDC (86.7 vs. 87.5, *p* = 0.164) and Lysholm scores (90.3 vs. 91.3, *p* = 0.176) at 2‐year follow‐up, with no graft ruptures in either group [6]. In the revision setting, Huber et al. reported significant improvement in Lachman test results from preoperative values (*p* < 0.001) in 28 patients, with a re‐rupture rate of 7.1% [10]. In a separate comparison of 55 revision cases (28 RF vs. 27 HT), Huber et al. found no significant differences in IKDC, Lysholm, Tegner, or pain scores between groups [11].

**Table 3:** Clinical outcomes of the rectus femoris tendon graft.

Study	Year	*n*	Design	Procedure	Follow‐up	Key outcomes
Barroso et al. [3]	2026	211	Prospective case series	Primary ACL; ACL + ALL	NR	*Capsular violation 8.1%; graft amputation 2.4%; no extensor mechanism injury*
Rêgo et al. [34]	2024	80	Case series	Primary ACL	18 months	Lysholm 97.1; PASS 100%; graft failure 0%
Dhariwal et al. [6]	2026	40 RF vs. 40 HT	Prospective cohort	Primary ACL	2 years	IKDC 86.7 vs. 87.5 (ns); Lysholm 90.3 vs. 91.3 (ns); graft rupture 0%
Mahmud et al. [17]	2026	36	Retrospective cohort	Primary ACL	Mean 16.3 months	Lysholm 57.2 → 89.2 (12 mo), 94.6 (18 mo); VAS 3.2 → 0.7; 94.4% good/excellent at 12 mo
Raman et al. [30]	2026	50	Prospective cohort	Primary ACL	3 years	Lysholm 70.4 → 90.2 (3 yr); IKDC 57.3 → 82.0; Tegner 3.1 → 4.8; no graft stretching or laxity on stress radiographs
Huber et al. [10]	2024	28	Case series	Revision ACL	Mean 41.7 months	Lachman improved (*p* < 0.001); re‐rupture 2/28 (7.1%); donor‐site pain 3.6%
Huber et al. [11]	2025	28 RF vs. 27 HT	Matched cohort	Revision ACL	Min. 12 months	IKDC, Lysholm, Tegner: ns between groups
Rêgo et al. [32]	2025	31 RF vs. 44 HT	Cross‐sectional	Primary ACL	6 months	No difference in extensor performance between RF and HT (all *p* > 0.05)
Okutan et al. [22]	2026	54	Retrospective case series	Primary ACL	Mean 15.7 months	IKDC 84.1; Marx 7.1; ATTD 1.8 mm; extension LSI 96% at 60°/s; no selective RF muscle atrophy on MRI
Barros et al. [1]	2026	31	Retrospective case series	Primary ACL	6 months	Extension LSI 71% (*p* < 0.001); flexion LSI 93% (ns)
Osorio Salas et al. [23]	2026	25	Case series	ACL + ALL	12 months	Quadriceps deficit 1.3% at 6 months

*Note*: Key outcomes are italicised.

Abbreviations: ACL, anterior cruciate ligament; ALL, anterolateral ligament; HT, hamstring tendon; IKDC, International Knee Documentation Committee; NR, not reported; ns, not significant; PASS, patient‐acceptable symptom state; RF, rectus femoris.

Okutan et al. evaluated 54 patients at a mean follow‐up of 15.7 months after primary ACL reconstruction with a quadrupled RF autograft [22]. The mean IKDC score was 84.1 and the side‐to‐side anterior tibial translation difference was 1.8 mm, further supporting the safety and effectiveness of the RF tendon for primary ACL reconstruction at 1‐year follow‐up.

Mahmud et al. retrospectively evaluated 36 patients undergoing primary ACL reconstruction with a triple‐folded rectus femoris autograft (mean graft length 30.7 cm, mean diameter 8.2 mm) at a mean follow‐up of 16.3 months [17]. The mean Lysholm score improved significantly from 57.2 ± 7.1 preoperatively to 89.2 ± 2.5 at 12 months and 94.6 ± 1.3 at 18 months (*p* < 0.001), and the mean VAS pain score decreased from 3.2 to 0.7 over the same period. At 12 months, 94.4% of patients achieved good or excellent functional outcomes.

At the longest available follow‐up, Raman et al. prospectively assessed 50 patients 3 years after primary ACL reconstruction with a doubled or tripled superficial quadriceps tendon graft (graft length 28–30 cm, diameter 8.5–10 mm) [30]. The mean Lysholm score increased from 70.4 ± 8.5 preoperatively to 89.6 ± 8.4 at 1 year and was sustained at 90.2 ± 8.4 at 3 years; the mean Subjective IKDC score improved from 57.3 ± 8.1 to 84.5 ± 7.8 at 1 year and 82.0 ± 8.3 at 3 years; and the mean Tegner Activity Scale increased from 3.1 ± 1.3 to 4.8 ± 1.2 at 3 years. No clinically significant graft stretching or laxity was observed on serial stress radiographs.

### DONOR‐SITE MORBIDITY

Post‐operative donor‐site complications were infrequent and of low severity. Haematoma was reported in 5% of cases in one series [34] and 0.9% in another [3]. Superficial infection occurred in 1% [34], and prolonged donor‐site pain in 3.6% [10]. No patient required surgical intervention due to postoperative morbidity.

A relevant concern with the rectus femoris harvest is the potential impact on quadriceps strength. Barros et al. performed a detailed isokinetic evaluation of 31 patients at 6 months after primary ACL reconstruction with RF autograft [1]. The operated limb achieved only 71% of contralateral extension peak torque (*p* < 0.001) at 6 months, with significant deficits in all extensor parameters [1]. In contrast, flexor performance showed near‐complete recovery, with a limb symmetry index of 93% for peak torque and no significant difference in any flexor parameter [1]. In a secondary analysis of the same 31 patients from the RF group, Rêgo et al. compared 75 patients (31 RF vs. 44 HT) and found no difference in isokinetic extensor performance between groups [32]. This indicates that the extensor deficit observed after RF harvest is not greater than that produced by HT harvest, although both groups remain well below the 90% limb symmetry threshold at this timepoint. Osorio Salas et al. independently reported a quadriceps strength deficit of 13% compared with the contralateral limb at 3 months, decreasing to 1.3% at 6 months, in 25 patients undergoing combined ACL and anterolateral ligament reconstruction [23].

Okutan et al. provided the first comprehensive one‐year evaluation of donor‐site morbidity after RF harvest using quantitative magnetic resonance imaging and isokinetic testing in 54 patients [22]. Volumetric MRI analysis demonstrated that the volume deficits were uniformly distributed across all four quadriceps muscles—rectus femoris (9.6%), vastus lateralis (8.5%), vastus intermedius (8.0%) and vastus medialis (8.1%)—with no significant between‐muscle difference (*p* = 0.170), indicating that RF tendon harvest does not produce selective atrophy of the donor muscle. Isokinetic strength testing showed limb symmetry indexes of 96% for knee extension at 60°/s and 94% at 240°/s, and all hop test indexes exceeded 90%, meeting established return‐to‐sport thresholds. These findings represent the first 1‐year data on functional recovery after RF harvest and extend the evidence beyond the 6‐month timepoint reported by Barros et al. [1] and Rêgo et al. [32].

The apparent heterogeneity in donor‐site morbidity rates across studies (e.g., 71% LSI reported by Barros et al. [1] versus 1.3% deficit by Osorio Salas et al. [23]) reflects differences in metrics, timepoints, and patient populations. Barros et al. reported a limb symmetry index of 71% for isokinetic peak torque at 6 months after isolated ACL reconstruction, whereas Osorio Salas et al. reported a maximal strength deficit of 1.3% on manual dynamometry at 6 months after combined ACL and anterolateral ligament reconstruction. The 1‐year data from Okutan et al. [22] demonstrated an extension LSI of 94%–96%, indicating that progressive quadriceps recovery continues beyond 6 months and that early deficits are largely transient.

Mahmud et al. reported mild quadriceps weakness in 4 of 36 patients (11.1%) following primary ACL reconstruction with the rectus femoris autograft, which resolved with physiotherapy [17].

In a prospective cohort with three‐year follow‐up, Raman et al. observed no quadriceps contracture, an outcome attributed to the preservation of the deeper quadriceps layers when only the superficial layer is harvested [30].

### COMPLICATIONS

As with any graft harvesting technique, the rectus femoris harvest requires familiarity with the anatomy and surgical steps. Barroso et al. reported an overall rate of complications of 11.4% in a prospective series of 211 patients, including capsular violation (8.1%) and premature graft amputation (2.4%) [3]. Postoperative haematoma was reported in 0.9% of cases [3]. Most complications were minor, and capsular violations during graft initial dissection with scalpel did not result in intraoperative or clinical repercussions when repaired with Vicryl sutures [3]. The two most common technical errors were initiating dissection too close to the patella, where the tendon planes are less distinct with no clear cleavage plane, and premature graft amputation using the stripper before adequate mobilisation of 7–8 cm of dissected distal tendon, indicating the absence of residual adhesions to the remaining quadriceps mechanism [3]. In cases of premature graft amputation, a second RF graft was successfully harvested from the same donor site without the need to change the graft choice [3, 34]. In an earlier series of 80 patients, Rêgo et al. reported a graft amputation rate of 3.6%, with all cases occurring within the first 10 procedures [34]. No extensor mechanism rupture or neurovascular injury was reported in any published series [3, 10, 23, 34]. These findings indicate that the technique is reproducible, although complications are more frequent during the initial cases.

A learning curve is evident in the published data. Rêgo et al. [34] reported that all premature graft amputations occurred within the first 10 procedures, and Barroso et al. [3] observed that capsular violations were concentrated in the initial cases of each participating surgeon.

Mahmud et al. reported postoperative haematoma at the harvest site in 2 of 36 patients (5.6%), managed conservatively with ice application [17].

In a prospective cohort of 50 patients followed for three years, Raman et al. reported anterior knee pain in 16%, knee stiffness in 6%, and superficial infection in 1%; no graft stretching or clinically significant laxity was observed on serial stress radiographs [30].

## DISCUSSION

The rectus femoris tendon combines the length and adaptability of a long soft‐tissue graft with biomechanical properties equivalent to the patellar tendon, allowing combined intra‐ and extra‐articular reconstruction while preserving the hamstrings and the deeper quadriceps layers. However, it should not be viewed as a replacement for established grafts. It represents an additional option that expands the available reconstructive strategies.

### Clinical Implications

Specific scenarios in which the RF may be advantageous include revision ACL reconstruction [10], combined ACL and anterolateral or anteromedial procedures using a single tendon [2, 33], patients in whom a small or unpredictable hamstring graft diameter is anticipated, combined ACL and medial ligament injuries in which preservation of the hamstrings is desirable for medial stability, posterior cruciate ligament reconstruction where graft length and diameter are critical [19] and multiligament knee injuries, particularly in settings with limited access to allografts [9]. These indications are supported by anatomical and biomechanical data but require confirmation in high‐quality clinical studies.

### Limitations

Several limitations of the current evidence should be acknowledged. All available clinical studies are level III or IV, and no randomised controlled trial has been published. The maximum mean follow‐up reported is approximately 3.5 years in a revision series of 28 patients [10], while the longest follow‐up in primary reconstruction is 3 years [30]. These timeframes are insufficient to draw conclusions on re‐rupture rates or long‐term joint health. Return to sport has not been evaluated using objective criteria such as hop tests or standardised clearance protocols. Quadriceps strength has been compared with hamstring tendon grafts at 6 months, and the long‐term impact of the harvest on extensor function remains unknown. No study has assessed graft maturation using magnetic resonance imaging. Female patients are underrepresented in the published series. The majority of published clinical data originates from a few research groups, and independent validation with larger sample sizes is needed.

Future research should address these gaps with specific study designs. A multicenter randomised trial comparing RF and HT grafts in primary ACL reconstruction with a minimum 2‐year follow‐up and re‐rupture as the primary endpoint would provide the highest level of evidence. Long‐term isokinetic evaluation of quadriceps strength beyond 12 months is needed to clarify whether functional recovery is sustained over time. Magnetic resonance imaging studies should assess graft maturation and tunnel integration. A formal analysis of the learning curve using sequential complication rates would help define the training requirements for safe adoption. Finally, future studies should include adequate representation of female and adolescent patients, who are at higher risk of graft failure.

## CONCLUSION

The available evidence supports the rectus femoris tendon as a viable autograft for knee ligament reconstruction, with consistent anatomy, biomechanical properties within the range of established ACL grafts, and applicability to multiple clinical scenarios. Its length allows combined intra‐ and extra‐articular reconstruction from a single harvest while preserving both the hamstrings and the deeper quadriceps layers. Early clinical results are consistent across independent centres, with functional outcomes comparable to HT grafts, low donor‐site morbidity, and a reproducible technique. Comparative studies with adequate follow‐up are needed to confirm these results. The rectus femoris tendon should be considered in individualised graft selection for knee ligament reconstruction.

## AUTHOR CONTRIBUTIONS

Bernardo Garcia Barroso had the idea for the article, performed the literature search and data analysis, and drafted the manuscript. Márcio Cabral Fagundes Rêgo, Marcelo Cabral Fagundes Rêgo, Felipe Silveira Martins, Sergio Marinho de Gusmão Canuto, Marcos Barbieri Mestriner, Carlos Eduardo Franciozi and Camilo Partezani Helito critically revised the work for important intellectual content. All authors read and approved the final manuscript.

## CONFLICT OF INTEREST STATEMENT

The authors declare no conflicts of interest.

## FUNDING INFORMATION

The authors have no funding to report.

## ETHICS STATEMENT

The authors have nothing to report.

## Supporting information

Supporting File

## Data Availability

The authors have nothing to report.
