# Supplementary material for: Beyond the quadriceps tendon: The rectus femoris as a distinct autograft for knee ligament reconstruction—A narrative review
Source: J Exp Orthop. 2026 Jul 30;13(3):e70862. doi: 10.1002/jeo2.70862 (PMC13420839; doi:10.1002/jeo2.70862)
Supplement: Supplementary file 1 — Supporting File [file JEO2-13-e70862-s001.docx]

**Supplementary Table S1.** Summary of the 35 studies addressing the rectus femoris tendon as an autograft for knee ligament reconstruction, grouped by study type.

| **Reference** | **Study design** | **N** | **Focus / Procedure** | **Follow-up** | **Key findings** |
| --- | --- | --- | --- | --- | --- |
| **Anatomical studies (n = 3)** | | | | | |
| **Iriuchishima et al. [13]** | Cadaveric | 16 formalin-fixed knees | Anatomical evaluation of the rectus femoris tendon and related structures | — | *Mean RF tendon length 27.3 cm; narrowest width 15.3 mm. First detailed anatomical study confirming feasibility for graft harvest.* |
| **Marot et al. [18]** | Cadaveric | 10 specimens | RF tendon morphometry and practical landmarks for harvesting | — | *Mean length 30.1 cm; diameters 8.8 / 9.9 / 11.4 mm in 2/3/4-strand. Two fusion zones identified (22 mm distal and 58 mm proximal), defining a 36-mm harvest window.* |
| **Thamrongskulsiri et al. [38]** | Cadaveric | 16 knees | Graft dimensions of superficial QT layer with minimally invasive harvest | — | *Mean graft diameter 9.7 mm (triple-strand); mean post-folding length 9.3 cm. No significant difference between sexes.* |
| **Biomechanical studies (n = 6)** | | | | | |
| **Chivot et al. [4]** | Cadaveric biomechanical | 10 fresh-frozen cadavers; 76 specimens tested (~7 per group) | Tensile properties of each QT layer (central and medial portions) | — | *Superficial QT layers (RF-equivalent) showed mean ultimate stress 75–95 MPa, significantly superior to iliotibial band (23 MPa) but lower than patellar tendon (141 MPa); comparable to two-strand hamstring (99 MPa).* |
| **Egiazaryan et al. [8]** | Cadaveric biomechanical, controlled | 24 donors / 24 specimens (n = 8 per group; 3 groups) | Mechanical strength of QT grafts (superficial-layer vs full-thickness vs peroneus longus) | — | *Superficial-layer QT (RF-equivalent) had the highest ultimate load (1043 ± 4 N), stiffness (156 ± 13 N/mm), and elastic modulus (94.5 ± 8.6 MPa), significantly exceeding both full-thickness QT (728 ± 39 N; 84 ± 15 N/mm; 52.8 ± 9.4 MPa) and peroneus longus (908 ± 74 N; 101 ± 12 N/mm; 78.2 ± 9.2 MPa) (all p < 0.001). Elongation at failure lowest for superficial-layer QT (23.5%) vs full-thickness QT (26.6%) vs peroneus longus (35.9%).* |
| **Gadelha Lopes et al. [9]** | Cadaveric biomechanical | 6 donors / 12 knees / 58 grafts (n = 6 doubled RF; 7 graft configurations) | Comparative biomechanical strength of 7 autograft configurations | — | *No significant difference in ultimate load among doubled RF (1714 ± 56 N), patellar tendon (1735 ± 136 N), and quadruple parallel hamstring (1684 ± 81 N). Full-thickness QT highest (2303 ± 80 N); iliotibial band lowest (749 ± 155 N).* |
| **Mestriner et al. [20]** | Cadaveric biomechanical, paired | 8 paired fresh-frozen knees (n = 8 per graft type; 32 total) | RF tendon for combined ACL + ALL biomechanical evaluation | — | *Doubled RF (1978 ± 338 N) had ultimate tensile strength equivalent to patellar tendon (1824 ± 557 N); both significantly higher than iliotibial band (819 ± 268 N; p < 0.01). Single-strand RF (1445 ± 584 N) showed biomechanical properties not different from ITB, supporting its use for lateral extra-articular reinforcement.* |
| **Pineda et al. [27]** | Paired cadaveric biomechanical | 7 paired organ donors (n = 7 per graft type; 14 grafts) | Doubled RF vs patellar tendon ultimate stress | — | *Doubled RF showed comparable ultimate stress to patellar tendon (46.4 ± 10.5 vs 52.9 ± 9.7 MPa, p = 0.184) despite lower load-to-failure (886 ± 52 vs 1279 ± 208 N, p < 0.001) and greater elongation at failure (1.2 ± 0.2% vs 0.2 ± 0.1%, p < 0.001).* |
| **Zhu & Zhang [42]** | Cadaveric anatomical + biomechanical | 10 cadavers / 20 knees (anatomy); 5 cadavers / 10 RF samples (biomech) | RF tendon anatomy and tensile properties vs native ACL | — | *Mean RF tendon length 6.96 ± 0.80 cm; width 3.20 ± 0.33 cm at patellar insertion. Single-strand RF tensile properties (unit modulus, maximum load, ultimate stress) statistically not different from native ACL.* |
| **Surgical technique descriptions (n = 14)** | | | | | |
| **Barroso et al. 2024 [2]** | Surgical technique description | — | Combined ACL + ALL reconstruction with superficial layer QT graft | — | *First step-by-step description of combined intra- and extra-articular reconstruction (ACL + ALL) using a single RF/superficial QT autograft.* |
| **Cury et al. [5]** | Surgical technique description | — | Double-bundle PCL reconstruction with superficial QT + semitendinosus | — | *Technique for double-bundle PCL combining the RF/superficial QT (anterolateral bundle) and semitendinosus (posteromedial bundle).* |
| **Di Muro et al. [7]** | Surgical technique description | — | Combined ACL + ALL reconstruction using RF tendon | — | *Detailed surgical technique for combined ACL + ALL with RF autograft.* |
| **Lee et al. [15]** | Surgical technique description | — | Bidirectional tendon strip technique for RF graft harvest | — | *Bidirectional harvest combining proximal and distal approaches for improved control during dissection.* |
| **Lyra de Oliveira et al. [16]** | Surgical technique description | — | Combined ACL + ALL with RF graft, single femoral tunnel + adjustable cortical suspensory fixation | — | *Technique using a single femoral tunnel with adjustable cortical suspensory fixation for combined ACL + ALL reconstruction.* |
| **Martins et al. [19]** | Surgical technique description | — | Double-bundle PCL reconstruction with RF/superficial QT graft alone | — | *Y-shaped configuration of the RF tendon for double-bundle PCL reconstruction.* |
| **Morin et al. [21]** | Surgical technique description | — | Continuous bone–QT–RF autograft for revision ACL + ALL | — | *Composite graft (patellar bone block + partial-thickness QT + RF) for revision ACL + ALL, optimizing tibial fixation in cases with tunnel widening.* |
| **Pettinari et al. [24]** | Surgical technique description | — | Revision ACL + lateral extra-articular procedure with RF autograft | — | *Technique description for revision ACL combined with lateral extra-articular reinforcement using RF autograft.* |
| **Pires et al. [28]** | Surgical technique description | — | Minimally invasive QT/superficial layer harvest | — | *Minimally invasive approach for QT/superficial layer harvest.* |
| **Quyen et al. [29]** | Surgical technique description | — | Superficial QT autograft harvest with transverse skin incision | — | *Transverse skin incision approach for the superficial QT/RF harvest.* |
| **Raman et al. 2022 [30]** | Surgical technique description | — | Minimally invasive and simple superficial QT graft harvesting | — | *First minimally invasive technique for superficial QT harvest using closed tendon stripper; foundational technique paper.* |
| **Rêgo et al. 2025 [33]** | Surgical technique description | — | Combined ACL + anteromedial oblique ligament reconstruction with RF tendon | — | *Technique for combined ACL + AOL reconstruction using the RF tendon, extending the principle of combined intra-/extra-articular reconstruction to the medial side.* |
| **Sonnery-Cottet et al. [36]** | Surgical technique description | — | Quad 2.0 — single RF autograft for combined ACL + double-bundle ALL | — | *Single RF graft used for combined ACL with double-bundle ALL reconstruction.* |
| **Thamrongskulsiri et al. 2023 [37]** | Surgical technique description | — | Minimally invasive harvest of triple-fold superficial QT autograft | — | *Triple-fold superficial QT configuration with tactile confirmation of the dissection plane.* |
| **Clinical studies (n = 11)** | | | | | |
| **Barros et al. [1]** | Retrospective case series | 31 patients | Isokinetic evaluation of extensor and flexor performance after RF ACL reconstruction | 6 months | *Extension LSI 71% (p < .001) at 6 months; flexor LSI 93%, no significant difference. Significant extensor deficit at 6 months; flexor performance near-complete recovery.* |
| **Barroso et al. 2026 [3]** | Prospective multicenter case series | 211 patients | Anthropometric prediction of graft dimensions and surgical pearls | Intraoperative | *Mean tendon length 30.8 cm; diameter ≥ 8 mm in 98.6% of triple-strand grafts. Height strongest predictor of tendon length. Capsular violation 8.1%, premature graft amputation 2.4%, hematoma 0.9%; all complications minor.* |
| **Dhariwal et al. [6]** | Prospective comparative cohort | 40 RF vs 40 HT | Functional outcome — superficial QT vs hamstring autograft | 2 years | *IKDC 86.7 vs 87.5 (p = 0.164, ns); Lysholm 90.3 vs 91.3 (p = 0.176, ns); no graft ruptures in either group.* |
| **Huber et al. 2024 [11]** | Retrospective case series | 28 patients | Revision ACL reconstruction with isolated RF autograft | Mean 41.7 months | *Significant Lachman improvement (p < 0.001); re-rupture rate 7.1%; prolonged donor-site pain 3.6%. First clinical revision series. Original technique description.* |
| **Huber et al. 2025 [12]** | Retrospective matched cohort | 28 RF vs 27 HT | Revision ACL — RF vs hamstring autograft | Minimum 12 months | *No significant differences between RF and HT for IKDC, Lysholm, Tegner, or pain scores.* |
| **Mahmud et al. [17]** | Retrospective cohort | 36 patients | Short-term follow-up of ACLR with triple-folded RF autograft | Mean 16.3 months | *Lysholm 57.2 → 89.2 (12 mo), 94.6 (18 mo); VAS 3.2 → 0.7; 94.4% good/excellent at 12 mo. Mild quad weakness 11.1% (resolved with PT); hematoma 5.6%.* |
| **Okutan et al. [22]** | Retrospective case series | 54 patients | Donor-site morbidity at 1 year (qMRI, isokinetic, hop tests) | Mean 15.7 months | *Uniform quadriceps volume loss (RF 9.6%, VL 8.5%, VI 8.0%, VM 8.1%; p = 0.170). Extension LSI 96% (60°/s) and 94% (240°/s); hop tests > 90%. IKDC 84.1; Marx 7.1; ATTD 1.8 mm.* |
| **Osorio Salas et al. [23]** | Surgical technique + case series | 25 patients | Open RF harvest technique + donor-site strength evaluation in combined ACL + ALL | 12 months | *Mean quadriceps strength deficit decreased from 13% at 3 months to 1.3% at 6 months and resolved by 12 months; supports rapid recovery profile with the open harvest approach.* |
| **Raman et al. 2026 [31]** | Prospective cohort | 50 patients | Functional outcome ACLR with superficial QT — 3-year follow-up (longest in this review) | 3 years | *Lysholm 70.4 → 89.6 (1 yr) → 90.2 (3 yr); IKDC 57.3 → 82.0; Tegner 3.1 → 4.8. No graft stretching or laxity on stress radiographs. Anterior knee pain 16%; stiffness 6%; superficial infection 1%.* |
| **Rêgo et al. 2024 [32]** | Retrospective case series | 80 patients | First clinical series of primary ACL reconstruction with RF | 18 months | *Mean Lysholm 97.1; 100% PASS; hematoma 5%; superficial infection 1%; graft amputation 3.6% (all in first 10 procedures, indicating learning curve).* |
| **Rêgo et al. 2025 [34]** | Cross-sectional comparative | 31 RF vs 44 HT | Isokinetic extensor and flexor performance — RF vs HT | 6 months | *No difference in isokinetic extensor performance between groups; both below 90% LSI threshold at 6 months. RF harvest does not produce greater extensor deficit than HT harvest.* |
| **Editorial / Expert commentary (n = 1)** | | | | | |
| **Pineda et al. [26]** | Editorial | — | Rectus femoris tendon as emerging option for ACL reconstruction | — | *Expert commentary highlighting current state of RF tendon use in knee ligament reconstruction and identifying research priorities.* |

*ACL, anterior cruciate ligament; ALL, anterolateral ligament; AOL, anteromedial oblique ligament; ATTD, anterior tibial translation difference; HT, hamstring tendon; IKDC, International Knee Documentation Committee; ITB, iliotibial band; LSI, limb symmetry index; PASS, patient-acceptable symptom state; PCL, posterior cruciate ligament; PT, physiotherapy; QT, quadriceps tendon; RF, rectus femoris; UTS, ultimate tensile strength; VAS, visual analog scale; VL, vastus lateralis; VI, vastus intermedius; VM, vastus medialis; ns, not significant; NR, not reported.*
